# 

**Published:** 1889-03-02

**Authors:** 


**Notice to Advertisers and Subscribers.**

All Advertisements, Orders for Copies of The Hospital, and Business Communications should be addressed to The Publisher, *not to the Editor*, The Hospital, 38, Old Jewry, E.C.

**To Residents, Secretaries, and Matrons.**

The Editor will be glad to have the Regulations and each Report as issued by Asylums, Hospitals, Convalescent and Nursing Institutions, as well as those of other Charities, and an early copy in duplicate of all Appeals and Festival Notices, etc., addressed to *The Editor, The Lodge, Porchester Square, London, W.* Any superintendent, secretary, matron, or other chief official who regularly sends such information may be registered, on application to the Publisher, to receive free of cost a cover for binding The Hospital in half-yearly volumes.

A Scale of Charges will be found on the first page of the Nursing Supplement.

### Scraps and Gleanings.

Mr. Keiller, of marmalade fame, has given £105 to the Aberdeen Royal Infirmary.

It is proposed to establish a district nurse in connexion with Swindon Victoria Hospital.

Lady Dufferin has consented to become a patroness of the New Hospital for Women.

There were thirteen applications for the vacancy at the Dublin Hospital for Incurables.

The Bishop of Manchester has started a discussion on rate-supported hospitals in the local press.

Mr. T. Mayo's tender of £4,715 has been accepted for the New Workhouse Infirmary at Coventry.

The Rev. Professor Chapman presided at the annual meeting of the Plymouth Homœopathic Hospital.

Prince Albert Victor will preside at the dinner in aid of the building fund of the Great Northern Hospital.

Portraits of Sir William Jenner and Sir George Burrows have been presented to the Royal College of Physicians.

There is still a good deal of irritation felt in local circles about that white elephant, the Mortlake Infectious Hospital.

Stockton Hospital has nominated a committee which is trying to establish the penny-a-week plan of workmen's subscriptions.

The Liverpool Consumption Hospital has reduced its charges to 2s. 6d. and 7s. a-week for nominated and unnominated patients respectively.

The Ulster Hospital for Women and Children issues a good report. The lady probationer system of nursing is found to work satisfactorily.

Mr. F. T. Barry, of St. Leonard's Hill, has given £1,000 to the Windsor Royal Infirmary for the erection of an accident ward at that institution.

The new infectious hospital for Wakefield has been opened. It contains four wards to hold sixteen beds, and cost £3,200. Mr. Watson was the architect.

The Tuesday entertainment at Brompton Hospital last week was given by Mrs. Edmund Tattersall assisted by Mr. John Thomas (harpist to the Queen).

The balance-sheet of Deptford and New Cross Provident Dispensary shows receipts amounting to £590, of which £516 is made up of members' subscriptions.

Mr. William Beer has been presented with a service of plate and a purse of sovereigns on resigning his post of secretary to the Royal South Hants Infirmary.

Sir Vincent Barrington presented the certificates and medallions to the successful pupils of St. John's Ambulance Association at Coventry on February 5th.

Mr. Percy S. Jakins, M.R.C.S., has been appointed surgeon to the Central London Throat and Ear Hospital, Gray's Inn Road, late senior assistant-surgeon.

Walsall Cottage Hospital issues a good report in spite of the strictures on its Sister Dora system of surgery, which have been expressed pretty freely lately.

Shrewsbury Hospital Saturday Fund has been awarded as follows: To the Eye, Ear, and Throat Hospital, £45; the Infirmary, £35; and to the Dispensary, £20.

At the annual meeting of the Cumberland Infirmary supporters on February 7th, the Bishop of Carlisle recommended the penny-a-week plan of raising subscriptions.

Bucks General Infirmary is always crowded with out-patients on Wednesday, which is market day. It is proposed to put on another dispenser for that day only.

A football-player named Knowlson, who got his leg broken in a match at Leek a fortnight ago, died in the Cottage Hospital, lockjaw having followed amputation.

A child aged two and a-half years, has died at Blackburn from the effects of being bitten by a rat. At Belfast a little girl has just died from the effects of the bite of a cat.

The Council of King's College have established a Chair of Neurology, to which Professor Ferrier has been elected. We understand that it is proposed to establish a neurological laboratory.

The Russian Red Cross Society have raised a large sum of money by selling envelopes stamped with their sign as a cover for New Year's cards. The *Daily News* recommends the idea to English charities.

A Medical Commissionership of Lunacy will shortly become vacant, by the resignation, through ill health, of Dr. Rhys Williams. The post, which is worth £1,500 a-year, is in the gift of the Lord Chancellor.

The first annual meeting since the amalgamation of Mansfield's two hospitals, took place on February 12th. The report submitted was satisfactory, and the building of the required extension will shortly be commenced.

Dolgelly boasts the lowest death-rate in England or Wales At a meeting of the sanitary body, Dr. Edward Jones, medical officer, reported that the death-rate for the past year was 13 per 1,000, which was the lowest on record.

It has been decided by the Correctional Court at Nice, that the London doctor who eloped to Paris with the young American lady must be set at liberty. Inasmuch as she was over sixteen years of age no charge could be preferred against him under French law.

Under the new scheme for the administration of the Army Medical Department, the office of Medical Director-General will in all probability be abolished, in which case the department will be directed by a board of three officers. Major-General Sir Redvers Buller, V.C., is mentioned as chairman of the board.

The following gentlemen, who competed at the examination recently held at Burlington House for appointment as surgeon in the Royal Navy, have been granted commissions:—O. W. Andrews, M.B., C. J. S. Kelsall, E. T. Cook, E. E. Powell, L. Bidwell, E. H. McSherry, W. J. Bearblock, W. Hackett, M.D., C. G. Matthews, M.B., J. Grant, M.B., G. A. Holroyd, T. Austen, and J. Chambers, B.A., M.B.

### Amusements and Relaxation.

### NEW PUZZLE PRIZE SCHEME.

**SECOND QUARTERLY COMPETITION COMMENCED JANUARY 5th, ENDS MARCH 30th, 1889.**

1. Prizes will be awarded for the best Original Puzzles in Prose or Verse relating to any of the Social, Political, or Philanthropical topics of the day. Competitors are requested to *strictly* observe this rule, or their contributions will be discredited.
2. Puzzles may take any form which the ingenuity of the competitors suggest. Answers must accompany contributions, and the source of obscure or ambiguous terms be given.
3. No competitor will be allowed to take more than one prize during the Quarter.
4. Eight Prizes of Standard Books will be given, the choice of the book to be left to the winner. First, Second, Third, Fourth, and Fifth Prizes for best Original Puzzles, value £1 1s., 15s., 10s. 6d., 7s. 6d., and 5s. each.
5. First, Second, and Third Prize for Solutions of 15s., 10s. 6d., and 5s. each.

N.B.—All contributions must reach the office, 38, Old Jewry, E.C., not later than the *First Post* on *Thursday* in each week.

### PUZZLES FOR SOLUTION.

**Ninth Week.**

No. 19. Double Acrostic.

My *primals* gave the people of my *finals* quite a scare,
That such things happen in our Isle most men were not aware.

1. Within the world the presence of this light we all deplore,
Against its power philanthropists still labour as of yore.
2. To those of various sects, of value small or great,
Some call "Divine," whilst others doubt the facts I state.
3. Where'er *I* abound
Desolation is found.
4. In hotter climes and under summer skies,
This plant is found whose root yields brilliant dyes.
5. How famous is this place most men can tell, and why?
Because its name is written on luxuries they buy.
6. Life is too sweet for most to wish for me,
Tho' e'en "a bare bodkin," the fettered soul would free.
7. Here the name of a state across the ocean wide,
Most curious customs here prevail, to one man many a bride.
8. Prove this suspected captive, and once more you are free;
The culprit must be sought for, this man it cannot be.
9. Of all my kind superbest, I flash with lustre rare;
No Eastern monarch's jewels can with me e'er compare.
10. An animal whose lovely fur, white as driven snow,
Adorns the cloaks and gowns of those whose dignity we know.

Cotonopolis.

No. 20. Enigma.

I cheer the palace and the cot,
Enliven all man's chequered lot;
Wealth and power immense I give,
No feeling have yet still I live;
Though short my life yet I supply
A thousand blessings ere I die.

Nipper.

**Marks for Original Puzzles.**

| Names. | No. of Puzzles sent in Feb. 21. | No. of Marks Feb. 21. | Totals. | Names. | No. of Puzzles sent in Feb. 21. | No. of Marks Feb 21. | Totals. |
|---|---|---|---|---|---|---|---|
| Cotonopolis... | 1 | 3 | 34 | Prior............ | 3 | 3 | 12 |
| Tinie............ | 3 | 4 | 35 | Lightowlers . | 3 | 5 | 18 |
| Jenny Wren . | 1 | 0 | 4 | Twentieth ... | — | — | 3 |
| Patience ...... | 3 | 6 | 40 | Crocodile...... | — | — | 5 |
| Padre ......... | — | — | 3 | Nabob ......... | 1 | 3 | 5 |

**Answers.**

No. 14. Triple Acrostic.

| | | | | |
|---|---|---|---|---|
| E | ra | S | in | G |
| N | ove | L | ett | E |
| G | re | A | te | R |
| L | exo | V | oru | M |
| A | lg | E | ri | A |
| N | a | R | a | N |
| D | r | Y | l | Y |

No. 15. Original Anagram.

"The Prosecution of the Bishop of Lincoln."

No. 16. Square Word.

S N O W
N E M I
O M E N
W I N D

**Marks for Solution of Puzzles.**

Total Marks.—Flora (2), 8; Mater (1), 4; Scrooge (2), 8; Patience (2), 4; Lady Clara (2), 7; Ruffles (2), 6; Lauriston, (1), 1; Reldas (2), 4; Jenny Wren (0), 2; Condorcet (1), 2; Tinie, 2.

[*The Figures in parentheses are Marks for the week ending Feb. 21st.*]

**Conditions.**

I. Every paper sent in must be clearly written, and one side only must be used. All competitors must mark at the head of their papers the number of their competition.

II. Each paper must be signed by the author with his or her real name and address. A *nom de plume* may be added if the writer does not desire to be referred to by us by his real name. In the case of all prizewinners, however, the real name and address will be published.

III. All communications relating to prize competitions should be addressed to "The Prize Editor, The Hospital, 38, Old Jewry, London, E.C." Competitors are requested not to include in letters, etc., to the Prize Editor, communications on matters of other business. All letters must be fully prepaid.

IV. The Prize Editor of The Hospital is the sole judge of the competitions, and his decision is in all cases final and without appeal.

V. The Prize Editor reserves the right to publish any paper sent in, whether it receives a prize or not. He does not hold himself responsible for any MSS. sent, nor can he undertake to return rejected competitions.

